# The Influence of Season on Diseases in Buffalo

**Published:** 1884-10

**Authors:** F. R. Campbell

**Affiliations:** Lecturer on Hygiene, Niagara Medical College


### The Influence of Season on Diseases in Buffalo.

By F. R. Campbell, A. M., M. D.

Lecturer on Hygiene, Niagara Medical College.

Ever since the celebrated Arbuthnot, in 1733, published his article on "The Effects of Air on the Human Body," the influence of season on disease has been quite generally recognized. The difficulties attending an accurate investigation of this subject are quite manifest. Not only do atmospheric pressure, moisture and temperature, all of which influence the progress of disease, vary greatly in the different seasons, but in the same season of different years show remarkable differences. Sudden variations in temperature, which are extremely common in our climate, have far worse effects than uniform high or low temperature. A sudden fall of temperature is more dangerous than a rapid rise. Dr. Farr concludes that the danger of dying from a sudden decline of temperature doubles every nine years after a person has passed his twentieth year. Drs. Richardson and Farr have deduced, from the vital statistics of the United Kingdom, some very interesting conclusions regarding the relation of season to disease. In this State, and especially in Buffalo, vital statistics are of so recent a date that the results obtained are not so valuable as they would be could our observations extend over a greater number or years. In studying the relations of season to disease in Buffalo we have divided the year into quarters, observations being extended over a period of three years, from 1881 to 1883 inclusive.

First, in regard to general mortality. This is quite uniform during the first, second and fourth quarters, being 21.8, 21.1,

20.9 per 1,000 respectively. But there is a marked increase in the death rate in the third quarter, reaching 29 per 1,000, on an average, for the three years. The maximum death rate is reached in August. This remarkable increase in the death rate is due to the prevalence of bowel diseases in Buffalo during this period. In England the death rate is greatest in the fourth quarter, 28 per cent. of the deaths occurring during that period, while the minimum number occur during the second quarter.

In order to show the effects of seasons on the more common diseases, and on infant mortality, the following table has been constructed, showing the death rate per 1,000 during each of the four periods into which we have divided the year. Among zymotic diseases are included cholera infantum, dysentery and malarial diseases. In the class of constitutional diseases we include rheumatism, phthisis, cancer, etc.:

| Quarter. | Under 5 yrs. | Zymotic. | Constitutional. | Local. | Scarlet Fever. | Diphtheria. | Cholera Infantum. | Dysentery. | Pneumonia & Bron'tis. | Gene'l death rate. |
|---|---|---|---|---|---|---|---|---|---|---|
| 1st | 9.2 | 5.8 | 3.15 | 8.9 | 1.7 | 1.75 | .... | .... | 3.35 | 21.8 |
| 2d | 7.9 | 5.4 | 3.60 | 7.9 | 1.4 | .55 | 0.11 | 0.21 | 2.80 | 21.1 |
| 3d | 18.0 | 13.5 | 3.30 | 7.6 | 1.0 | .48 | 7.13 | 2.35 | 1.57 | 29.0 |
| 4th | 9.4 | 7.2 | 2.90 | 6.4 | 1.9 | 1.62 | .53 | 0.40 | 1.97 | 20.9 |
| Average | 11.1 | 8.0 | 3.24 | 7.7 | 1.4 | 1.10 | 2.00 | 0.75 | 2.68 | 23.2 |

In the first quarter pulmonary diseases and diphtheria are most prevalent, while intestinal diseases are almost unknown.

In the second quarter the deaths from constitutional diseases seem to be greater. Phthisis appears to be most fatal at this time. But in June the general death rate is lower than at any other period of the year.

In the third quarter there is a sudden increase of cholera infantum and dysentery, so remarkable that these diseases may be said to be confined to this period of the year. Scarlet fever, pulmonary diseases and diphtheria are now at their minimum.

In the last quarter scarlet fever is at its maximum, while diphtheria and respiratory diseases greatly increase.

We have constructed the following comparative table to show the periods of greatest and least intensity of the more common diseases observed in London, New York City and Buffalo:

| City. | Scarlet Fever. Maximum. | Scarlet Fever. Minimum. | Diphtheria. Maximum. | Diphtheria. Minimum. | Cholera Infantum. Maximum. | Cholera Infantum. Mini'm. | Pneumonia. Max'm. | Pneumonia. Mi'm. |
|---|---|---|---|---|---|---|---|---|
| London | Sept. to end of y'r. | Feb. to 1st of Aug. | Sept. to end of y'r. | March to Sept. | July & Aug. | March & April. | Dec. | Aug. |
| New York | April. | Sept. | Dec. | Aug. | July & Aug. | Feb & March. | March. | Aug. |
| Buffalo | Dec. | Sept. | Jan. | Aug. | Aug. | 1st q'r. | Feb. | Aug. |

On referring to the above table it will be observed that scarlet fever increases in severity later in this country than in England, while it assumes its mildest form in London in April, the very month when it is most severe in New York. Diphtheria is most severe in this State in the coldest months, while in England it is most fatal in the fall and early winter, a period characterized by dampness and cold. Pneumonia is also with us most fatal in the coldest months, and not most severe in damp weather, as in England. Cholera infantum and dysentery begin, as a rule, about a month later in Buffalo than in New York, or about the same time the disease is most prevalent in England.

